# Supplementary material for: “I could hang up if the practitioner was a prat”: Australian men’s feedback on telemental healthcare during COVID-19
Source: PLoS One. 2022 Dec 14;17(12):e0279127. doi: 10.1371/journal.pone.0279127 (PMC9749969; doi:10.1371/journal.pone.0279127)
Supplement: S1 Appendix — (DOCX) [file pone.0279127.s001.docx]

**S1 Appendix - Survey items**

**Demographics**

What is your age in years? Please just write the number: ______________

How would you best describe your sexual orientation?

- Straight
- Gay
- Bisexual
- I use another term: ______________

What is your highest level of completed education?

- Some high school
- Trade/certificate/diploma
- High school
- Undergraduate degree
- Postgraduate degree

How would you describe your current place of residence?

- Metropolitan
- Regional
- Rural or remote

What is your current employment status?

- Employed full time
- Employed part time
- Employed casually
- Unemployed, looking for work
- Unemployed, not looking for work
- Retired
- Student

**Help-seeking during pandemic**

Have you sought help from a mental health professional (i.e., psychologist, counsellor, or other therapist) since March 2020 (i.e., during the COVID-19 pandemic)?

- Yes
- No

Was seeking help since March 2020 (i.e., during the COVID-19 pandemic) **your first experience** seeing a mental health professional?

- Yes - seeking help in COVID was my first experience
- No - I had sought help prior to the pandemic

Once you made the decision to seek help from a mental health professional **during the COVID-19 pandemic**, approximately how long did you have to wait until your first session?

- A few days - 1 week
- 1-2 weeks
- 2 weeks - 1 month
- 1-2 months
- 2-4 months
- 4-6 months
- More than 6 months

**Experiences with telemental health**

Were some or all of your sessions conducted via telehealth (i.e., over the phone or via zoom/skype etc) in the past 18 months?

- Yes
- No (skip the next 5 questions)

**IF YES:** What proportion of your sessions with your most recent mental health practitioner were via telehealth?

- Less than half of them
- About half of them
- More than half of them
- All of them

**IF YES:** Do you prefer telehealth or in-person sessions?

- Definitely prefer telehealth
- Slightly prefer telehealth
- Neutral
- Slightly prefer in-person
- Definitely prefer in-person

**IF YES:** If you could continue to see a mental health practitioner via telehealth in future, would you?

- Definitely not
- Lean towards no
- Neutral
- Lean towards yes
- Definitely yes

**IF YES:** What did you **like** about seeing a mental health practitioner via telehealth (**what was good about it**)?

____________________________________________________________________

____________________________________________________________________

____________________________________________________________________

**IF YES:** What did you **not** **like** about seeing a mental health practitioner via telehealth (**what was bad about it**)?

____________________________________________________________________

____________________________________________________________________

____________________________________________________________________

Are you still working with your most recent practitioner?

- Yes
- No

**Satisfaction with therapy and therapist scale-revised**

Please circle the number that best describes your satisfaction with the therapy and therapist in your most recent experience.

Please respond using the scale from 1 (strongly disagree) to 5 (strongly agree).

1. I am satisfied with the quality of the therapy I received

2. The therapist listened to what I was trying to get across

3. My needs were met by the therapy

4. The therapist provided an adequate explanation regarding my therapy

5. I would recommend the therapy to a friend

6. The therapist was not negative or critical towards me

7. I would return to the clinic if I needed help

8. The therapist was friendly and warm towards me

9. I am now able to deal more effectively with my problems

10. I felt free to express myself in my therapy sessions

11. I was able to focus on what was of real concern to me

12. The therapist seemed to understand what I was thinking and feeling
