# Supplementary material for: “I could hang up if the practitioner was a prat”: Australian men’s feedback on telemental healthcare during COVID-19
Source: PLoS One. 2022 Dec 14;17(12):e0279127. doi: 10.1371/journal.pone.0279127 (PMC9749969; doi:10.1371/journal.pone.0279127)
Supplement: Attachment — Submitted filename: Mens experiences with telehealth_response to reviewers 2.docx [file pone.0279127.s002.docx]

| **Comment** | **Response** |
| --- | --- |
| Thank you for the opportunity to review this manuscript. Drawing on mixed methods research, this manuscript provides insights into the experiences of men accessing telemental health throughout the pandemic. The authors have done well to address previous reviewer comments. I have a few additional comments. | Thank you for the positive feedback, we have addressed each of your points in turn below. |
| Throughout: health care and healthcare appear to be used interchangeably throughout | Thank you for noting this – we have adjusted to refer consistently to “telemental healthcare” throughout. |
| Numbers below 10 should be written out (e.g., two, not 2) | These have been amended throughout where numbers refer to frequencies. In our measures section we have retained use of numbers for response scales, as the number was presented to participants in the scale in numerical form rather than word form (e.g., 1 – strongly disagree to 5 – strongly agree). |
| Abstract: How were these men recruited? | We have added a note to address this in the abstract:  *A community sample of Australian-based men (N=387; age M=47.5 years, SD=15.0 years) were recruited via Facebook advertising, and completed an online survey comprising quantitative items and open-response qualitative questions with the aim of better understanding men’s experiences with telemental healthcare services.* |
| Introduction: Page 3, lines 80-81: Feels like a qualifier is needed here. One in five Australian adults? | This has been amended as suggested. |
| Page 5, line 107: should this be “accompanying the COVID-19 pandemic”? | This has been amended as suggested. |
| Method: Page 6, line 129: Why 16+, rather than say, 18+? | We aimed to be inclusive of young men in our sampling. Our lower bound for age was set to 16, given young men aged under 18 but over 16 were considered mature minors, therefore able to provide their own informed consent to take part without the need for parental consent. We have added a note to address this in the relevant section as follows:  *Australian men aged ≥16 years (i.e., inclusive of mature minors) were invited to take part in a brief online survey about their mental health during the COVID-19 pandemic.* |
| Page 7, lines 160-161: was there much turnover of practitioner (and was this information collected)? | Unfortunately, this information was not collected. |
| Results: page 12, line 240: insert full stop | Thank you, this has been amended. |
| page 15, line 261 “on their own terms”: not clear what is meant by this - was it that they had the choice of attending in person or via telehealth? Or something else? | We have replaced this as follows to improve clarity:  *Fifty-five participants suggested that they engaged in telemental health treatment from the comfort of their own home or an environment of their choice, which resulted in a far less confronting experience relative to face-to-face healthcare interactions.* |
| Page 15, quote in lines 264-265: Is there more to this quote? It would seem that a therapist's office would likely have fewer external distractions than someone's home. | There was nothing else in this quote that was relevant to the sentiment being conveyed in this section – the full quote is as follows: “*Fewer external distractions. Can focus better. Sessions were focused upon the purpose for the consultation.*”  We agree that a therapists’ office could (and should) be expected to be less distracting than one’s home environment, however this did not seem to have been the case for this participant. Perhaps they found that the unfamiliarity of the therapists’ environment introduced novel distractions that did not affect their experience of therapy delivered via telemental health. |
| Page 16, lines 267-270 (quote): the quote seems more suited to the next sub-section – “Convenience and accessibility” | Thank you – we agree there is some overlap here. We hope to keep this quote in this section, as we feel this quote primarily conveys our findings regarding the comfort many participants experienced when accessing therapy remotely, with mention of accessibility accompanying this. |
| Pages 16-17, Lines 285 (“physical distance”) – 293: the next two quotes suggest it isn't the physical distance, but the anonymity of the phone (vs video telehealth sessions, such as over Zoom or FaceTime) that they appreciate. | Thank you, we acknowledge that we were unable to determine whether participants were referring to phone- or zoom-based telemental health appointments. We have removed the ‘physical distance’ aspect of this sentence in line with this. |
| Page 18, line 327: should “regards” be “regarding” or “with regards to”? | This has been amended to “regarding” as suggested. |
| Page 18, line 330: change "the frustrating" to "some frustrations with the". The way it is currently written suggests that the technical limitations are inherently frustrating, rather than only being frustrating for some people in some instances. | Thank you, this has been amended as suggested. |
| Page 19, line 343 “some”: Why is "some" used here when other responses are quantified with exact number of participants who relayed similar responses? | This has been amended to “four” participants. |
| Page 19, lines 345-356: This paragraph / section might be better placed after the one describing the pros of telehealth (can stay in pjs, don't need to travel, etc). | Thank you – we have opted to keep this paragraph in this section, such that all pros of telehealth are discussed as a main section, followed by all the cons in their own dedicated section. We acknowledge that the content in this paragraph is linked in meaning to the examples you mention, so we have added the following text to the beginning of the paragraph to provide linkage to the previous section:  *While accessing therapy from home presented advantages in terms of comfort for some participants, thirty-one participants spoke of the difficulty in navigating blurred boundaries between their private world (e.g., home and work) and the therapy space.* |
| page 22, line 402: “challenges being emotionally authentic remotely.” – is there a word missing? | There wasn’t a word missing, but we have amended this as follows to hopefully add clarity. By ‘remote’ we are referring to telemental health services (i.e., delivered remotely)  *Notwithstanding this, several telemental health shortfalls were also noted, themed around therapy interrupted and disconnection with service providers, blurred boundaries between home and therapy and challenges with remote displays of emotional authenticity.* |
| page 25, line 469 “utility of reflection”: is something missing here? e.g., utility of time/space for reflection, such as car drive home? | Thank you, this has been amended as follows:  *Ranging from difficulty finding a private area at home where they wouldn’t be disturbed or overheard, to missing the utility of time and/or space for reflection therapy (e.g., in the commute to or from therapy) posed challenges for some men.* |
| Limitations Page 27, lines 500-504: I think greater mention and exploration of this is needed in the discussion, particularly as the nuanced experiences of both are apparent in the data and results (e.g., page 19, line 348 – participant 179 quote and page 20, line 378, participant 110). | Thank you – this is a great point. We have added the following text to the discussion section just prior to discussing future implications:  *Moreover, apparent in our results were nuances regarding the benefits and drawbacks of telemental healthcare when delivered via videoconference (e.g., Zoom) or via phone. We were unable to determine whether participants were referring to videoconference or phone-based appointments in qualitative results (which is discussed below as a limitation). Our results nevertheless suggest that perhaps there are unique considerations for therapists when engaging men via videoconference relative to phone-based sessions. Videoconference sessions may allow therapists greater depth of communication via non-verbal cues, however this modality presents more technological frustrations due to reliance on internet connectivity. Conversely, phone-based sessions may alleviate technological concerns, but participants lamented their impersonal nature due to the lack of visual feedback. When delivering therapy via telemental health, gauging client preferences and aiming to cater to these where options for modes of service provision are available, could boost likelihood of engagement.* |
