# Supplementary material for: “I could hang up if the practitioner was a prat”: Australian men’s feedback on telemental healthcare during COVID-19
Source: PLoS One. 2022 Dec 14;17(12):e0279127. doi: 10.1371/journal.pone.0279127 (PMC9749969; doi:10.1371/journal.pone.0279127)
Supplement: Attachment — Submitted filename: Mens experiences with telehealth_response to reviewers.docx [file pone.0279127.s003.docx]

| **Comment** | **Response** |
| --- | --- |
| Reviewer #2: The authors have fully addressed all comments. I appreciate the explanations provided in instances where reviewer comments/suggestions were not implemented. | Thank you for the positive feedback, we appreciate the time taken to help us improve the manuscript. |
| My last remaining suggestion would be a sentence in the Methods "Experiences with telemental health" subsection, to explicitly state that in the survey, the term "telemental health" referred to both virtual (video) and phone (audio only). As this is addressed in the limitations, I think it is important for the reader to have this information up front prior to reading and interpreting the results. | Thank you – we have added the following sentence at the beginning of the “Experiences with telemental health” measures subsection to clarify this point:  *In this survey, the term ‘telemental health’ referred to both virtual (video) and phone-based (audio only) sessions.* |
